# Facile preparation and adsorption performance of graphene oxide-manganese oxide composite for uranium

**DOI:** 10.1038/s41598-018-27111-y

**Published:** 2018-06-13

**Authors:** Aili Yang, Yukuan Zhu, C. P. Huang

**Affiliations:** 1 Institute of Materials, China Academy of Engineering Physics, Jiangyou, 621907 China; 2 Department of Environmental Engineering, University of Delaware, Newark, DE 19716 USA

## Abstract

To overcome the limits of low adsorption capacity and the separation difficulty of solid from liquid phase for graphene oxide (GO), a novel nanocomposite graphene oxide-manganese oxide (GOMO) was facilely fabricated under ultrasonic radiation. The structures and micro-morphology of the products were characterized by fourier transform infrared (FT-IR) spectroscopy, raman shift spectroscopy, X-ray diffraction (XRD) pattern and scanning electron microscopy (SEM). The effect of solution pH, adsorbent dose, contact time, initial uranium concentration, ionic strength and temperature on uranium removal efficiency was studied by batch adsorption experiments. The product GOMO was used to examine the feasibility of the removal of high salt content in uranium-containing wastewater. The adsorption results were fitted using the Langmuir and Freundlich isotherm models. The kinetic parameters in the adsorption process were measured and fitted. Five adsorption/desorption cycles were performed using 3 M HNO_3_ as the regenerant in order to evaluate the reuse of GOMO.

## Introduction

The removal and recovery of nuclide uranium with a long half-life and hazardous radio-toxicity has been regarded as one of the most important and challenging research problems. With the rapid development of various activities related to uranium, most countries have established stringent guidelines for discharge of uranium into water. Therefore, the high-efficiency removal of uranium from aqueous solutions has become a hot research topic^1,2^. Sorption^3–5^, which is superior to other techniques (e.g., bioreduction and precipitation^6^, reverse osmosis^7^, and ion exchange^8,9^), has been widely applied in the wastewater treatment process due to its merits, such as economic, operation simplicity and no secondary pollution. However, most of adsorbents exhibit some disadvantages of low sorption capacity, high cost and lack of environmentally-friendly properties. Therefore, it is very necessary to explore the cheaper and more environmentally friendly adsorbents with higher sorption capacity to meet the current more stringent requirements of water quality and environment protection.

Recently, manganese oxides (Mn_x_O_y_) have attracted considerable attention owing to their excellent properties (e.g., abundance, environmental friendliness, cheapness and high stability. It has been proven that Mn_x_O_y_ has favourable adsorption action for the removal of heavy metal such as Pb^10^, Hg^11^, Sr and Co^12^. Nevertheless, Mn_x_O_y_ with fine particle sizes has limited practical application because of its slow solid-liquid separation. Therefore, it is necessary to decorate Mn_x_O_y_ with other large molecular compounds to obtain some superior properties and enhance the practical applications of Mn_x_O_y_^13^.

Graphene oxide (GO) with abundance of oxygen-containing groups (e.g., carboxyl and hydroxyl) has obtained plenty of attention owing to its large specific surface area, excellent adsorption performance and unique electronic properties^14,15^. However, it is well-known that some typical competing cations (e.g., Na^+^, K^+^, Mg^2+^, and Ca^2+^) commonly present in wastewater probably produce a certain interference for the adsorption capacity of GO as the adsorbent. Wan *et al*.^16^ reported a nanocomposite graphene oxide-manganese oxide with an outstanding sorption selectivity for Pb(II) when high amounts of Ca(II) coexisted. Moreover, the separation difficulty of the GO-loaded metal ions from liquid phase restricts the practical application of GO. Therefore, it is very valuable to prepare the composites of GO and other substance that combine their advantages. In our previous work we reported the preparation and adsorption performance of the composite GO and chitosan for uranium^17^. To our best knowledge, no studies have reported the fabrication of the composite GO/Mn_x_O_y_ under ultrasonic irradiation. Ultrasonic irradiation has been proven to be a highly efficient technique for nanocomposites synthesis because of its advantages of short time, low energy consumption and good shape and size control^18^.

**Figure 1:**
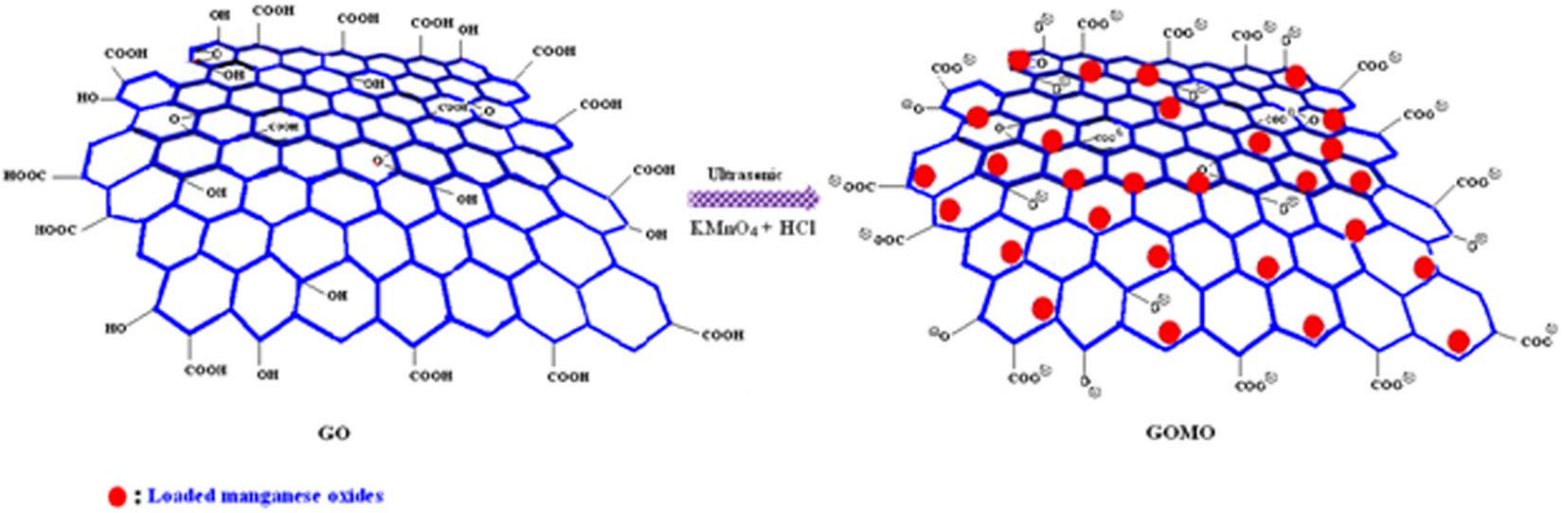
Schematic depiction of the formation of the composite adsorbent GOMO.

Herein, we modified the GO surface with Mn_x_O_y_ by the ultrasonic method and prepared the novel composite adsorbent graphene oxide–manganese oxides (GOMO). The preparation route of GOMO is shown in Fig. 1. The structure and micro-morphology of the products were characterized by FT-IR, raman shift and SEM. The influence of various factors such as pH, sorbent doge, adsorption time, initial uranium concentration, ions strength and temperature on the sorption behaviour of U(VI) onto GOMO from an aqueous solution was investigated. Moreover, the typical industry wastewater with high salinity has been discharged from the nuclear plant which results in the increasing difficulties of nuclear wastewater treatment. Therefore, the feasibility of the removal of high salt content in the wastewater samples using GOMO was also evaluated in this study.

## Results and Discussion

### Characterization

**Figure 2:**
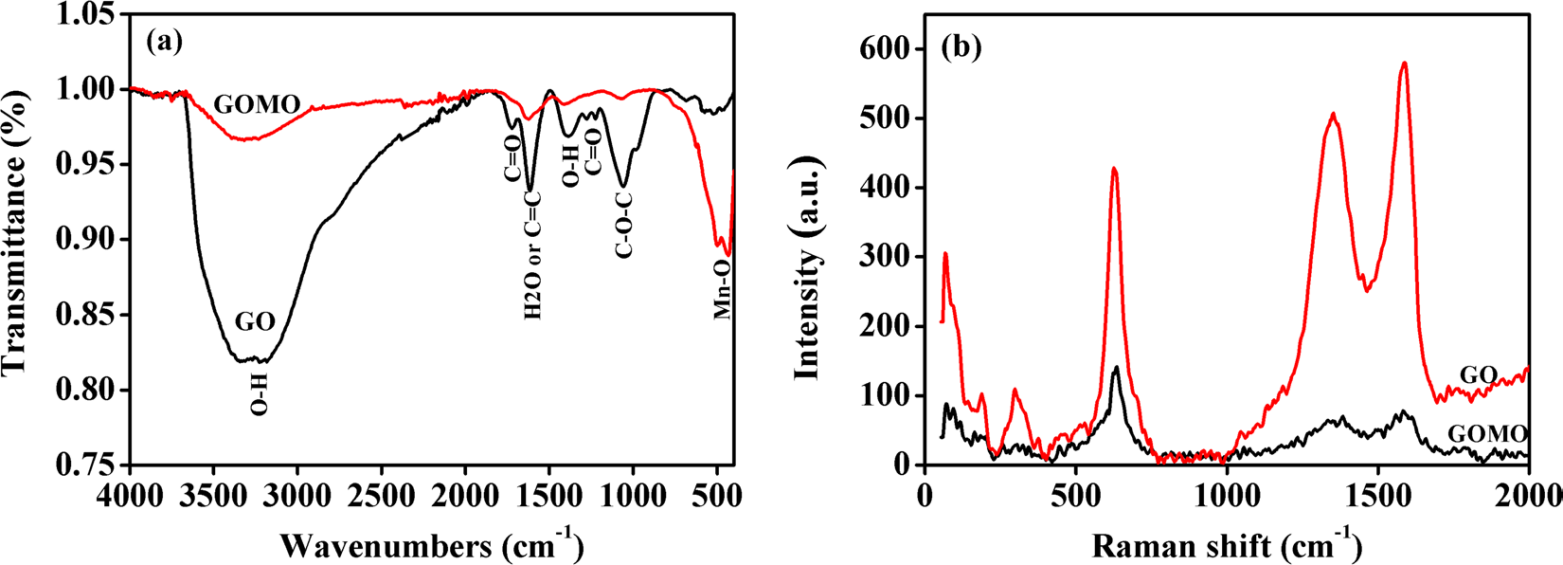
IR spectra (**a**) and raman shift spectra (**b**) of GO and GOMO.

The FT-IR spectra of GO and GOMO are given in Fig. 2(a). The IR spectrum of GO was similar to that of GO in the reference^19^ and showed characteristic peaks at 3345~3229, 1725, 1618, 1387, 1227 and 1061 cm^−1^, ascribed to O-H stretching vibration, C=O, aromatic C=C, O=C-O and C-O-C stretching vibrations, respectively. However, compared to GO, the characteristic peaks of GOMO assigned to O-H, C=O, C=C and C-O-C were shifted to 3321, 1626, 1413 and 1075 cm^−1^ and the relative peak intensities decreased and in some cases even disappeared. Moreover, the presence of a strong absorption peak at 501 cm^−1^ ascribed to Mn-O vibration and indicated that manganese oxides were loaded successfully onto the surface of GO.

The raman shift spectra of GO and GOMO are presented in Fig. 2(b). Compared to the band of GO, the intensity of the two characteristic peaks of G and D bands in the spectra of GOMO is significantly lower, indicating the formation of the chemical bonds between GO and Mn_x_O_y_.

**Figure 3:**
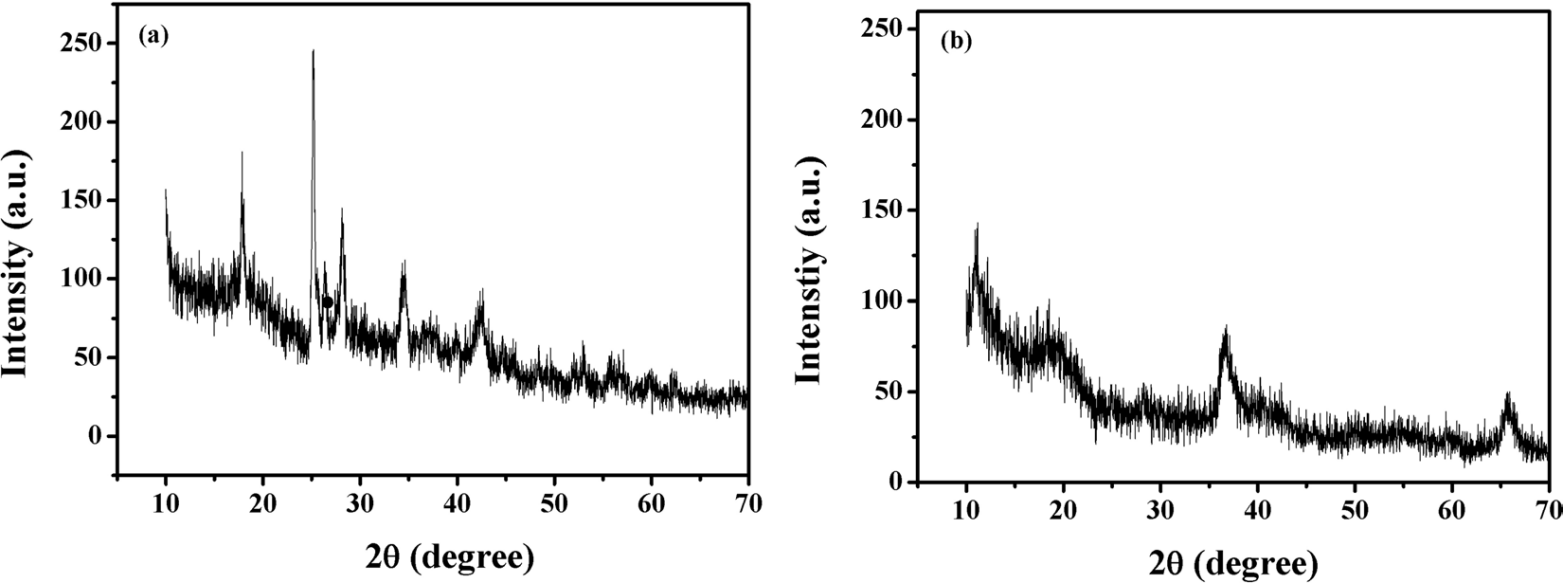
XRD patterns of GO (**a**) and GOMO (**b**).

In the XRD pattern of Mn_x_O_y_ it was clear that Mn_x_O_y_ was poor crystallized and two broad peaks were observed at 2θ values of 36.7° and 65.7°. Figure 3 showed the XRD patterns of GO (a) and GOMO (b). There were the significant difference between the XRD spectra of GO and GOMO. The XRD analysis of GOMO showed the intensity of all the peaks ascribed to that of stacked GO nanosheets reduced significantly with the increase of the Mn_x_O_y_ amount, suggesting that Mn_x_O_y_ was loaded in the surface of GO. Additionally, two feature diffraction peaks at about 2θ = 36.7° and 65.7° of Mn_x_O_y_ were detected on the composite GOMO (Fig. 3b), indicating the existence of Mn_x_O_y_ on GOMO. Furthermore, by EDS analysis the Mn mass was determined to be 17.40 wt% in GOMO while no Mn element was observed in GO which suggested that the successful combination between GO and Mn_x_O_y_.

**Figure 4:**
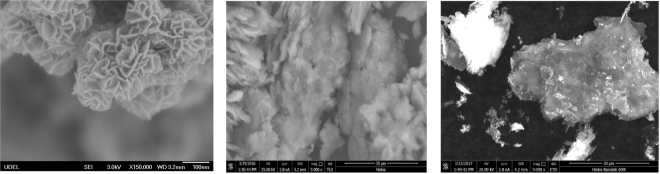
SEM images of Mn_x_O_y_ (**a**), GO (**b**) and GOMO (**c**).

Figure 4 shows that micro-morphologies of Mn_x_O_y_ (a), GO (b) and GOMO (c). Both GO and GOMO exhibited lamellar and wrinkled morphology. Moreover, it was seen from Fig. 4(c) that GOMO was covered by a large number of emerging flakes and particles, revealing that Mn_x_O_y_ were attached to the GO surface. Therefore, based on Figs 2, 3 and 4, we can conclude that the composite GOMO was successfully prepared.

### Sorption performance

#### Influence of solution pH on sorption efficiency

**Figure 5:**
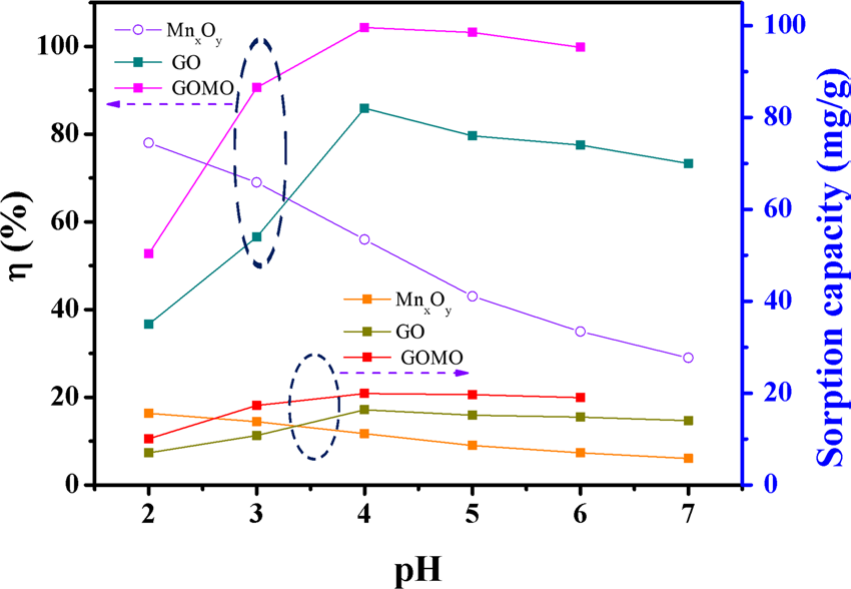
Influence of pH on the adsorption efficiency of uranium by Mn_x_O_y_, GO, and GOMO. *C*_0_(U) = 10 mg/l, adsorbent dosage = 0.5 g/l, T = 298 K, t = 30 min.

The removal efficiency of Mn_x_O_y_, GO and GOMO for U(VI) is presented in Fig. 5 at pH = 2.0–6.0. It was observed that pH had a remarkable influence on the adsorption of U(VI). The removal efficiency increased significantly with increased solution pH. The uranium removal rate of GO and GOMO reached the maximum value (nearly 100%) at pH 4.0. The results showed that the adsorption process depended strongly on the hydrolysed species of U at different pH. The predominant U form was UO_2_^2+^ at pH < 4.0, and the adsorption efficiency was low due to the competition between H^+^ and UO_2_^2+^ for the adsorption sites^20^. (UO_2_)_3_(OH)_5_^+^ become dominant at pH = 4.5–7.5^21^ which resulted in the significant increase of the adsorption efficiency due to electrostatic interaction between (UO_2_)_3_(OH)_5_^+^ and the GO and GOMO with negative charges. Meanwhile, the adsorption efficiency of GOMO was better than GO. The results indicated that the doping of Mn_x_O_y_ effectively enhanced the adsorption property of GO. Therefore, pH 4.0 was chosen as the optimum pH.

#### Influence of adsorbent dosage on sorption efficiency

**Figure 6:**
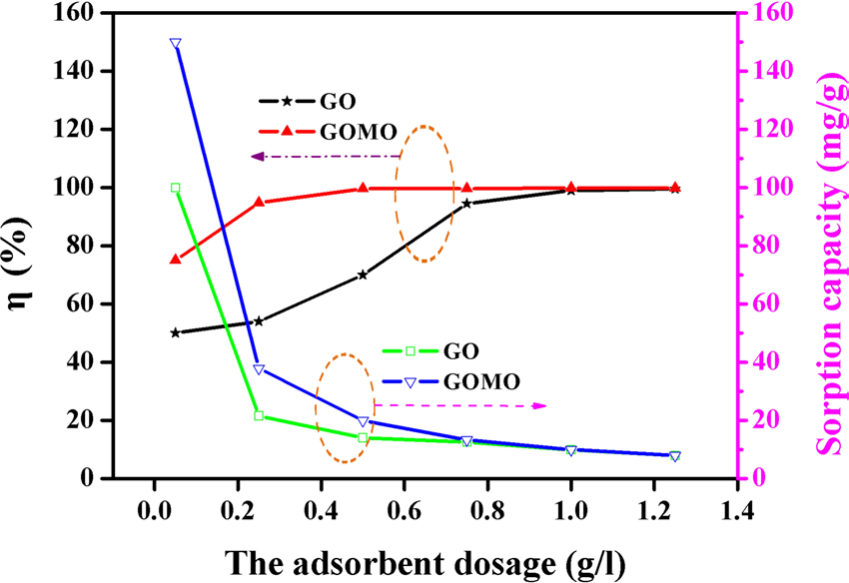
Influence of adsorbent dosage on the sorption efficiency of uranium by GO and GOMO. pH = 4.0, *C*_0_(U) = 10 mg/l, T = 298 K, t = 30 min.

The influence of adsorbent dosages on sorption efficiency is presented in Fig. 6. The removal efficiency increased sharply when low dosage was used, indicating that there were many readily accessible active sites. With further increase of the dosage, *Q*_e_ decreased significantly, while the removal efficiency of uranium shows a steady trend. The maximum removal rate of GO and GOMO reached above 99% when their dosage was 1.0 and 0.5 g/l, respectively. The reason might be fewer available active sites when the adsorption process completed which increases the difficulty of further loading of the adsorbent for uranium ions.

#### Influence of contact time and kinetic studies

**Figure 7:**
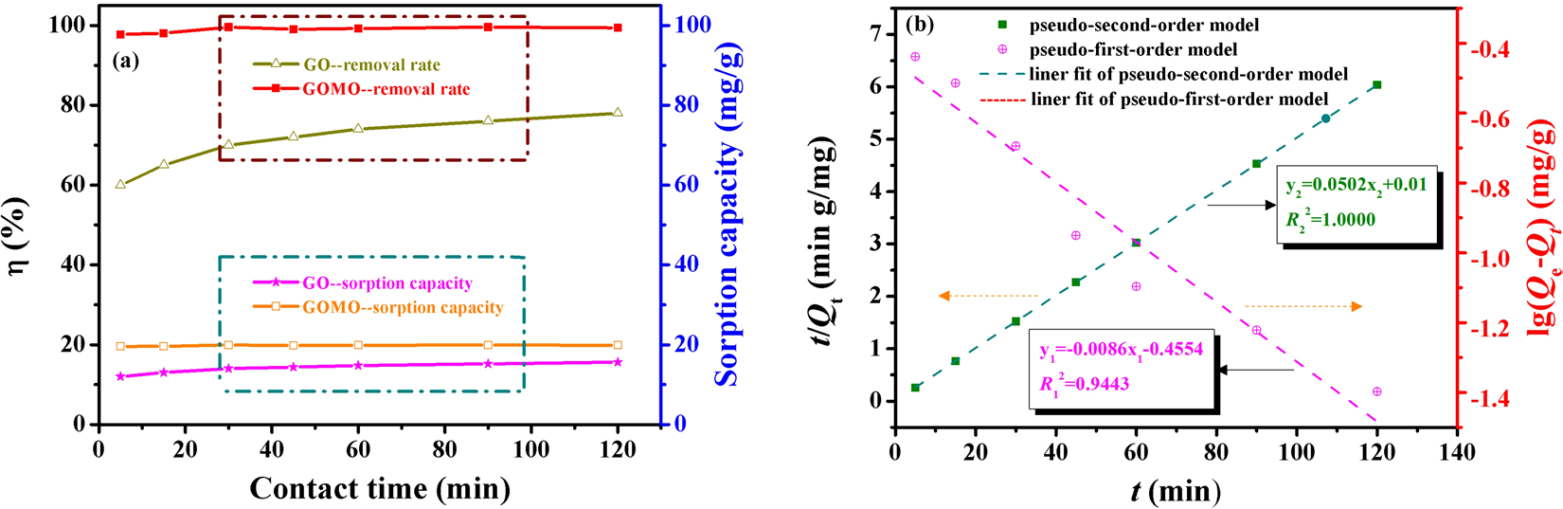
Influence of contact time on uranium sorption of GO and GOMO (**a**) and adsorption kinetics of U(VI) adsorbed by GOMO (**b**). pH = 4.0, *C*_0_(U) = 10 mg/L, adsorbent dosage = 0.5 g/L, T = 298 K.

Figure 7 presents the effect of contact time on the sorption of GO and GOMO, and linear fit of sorption kinetic of U(VI) adsorbed by GOMO. It was seen from Fig. 7(a) that GOMO reached favourable removal efficiency (nearly 100%) in a very short time. The sorption reached equilibrium when the surface active sites were saturated and hardly occupied. However, compared to GO the sorption efficiency of GOMO was evidently improved after the modification.

According to Eqs (9) and (10) the calculated parameters of *k*_1_, *k*_2_, *Q*_e_ and *R*^2^ are given in Table 1. As seen from Fig. 7(b) the sorption of U(VI) onto GOMO fitted the pseudo-second-order model well (*R*^2^ = 1.0000), suggesting that the adsorption of U(VI) onto GOMO was mainly controlled by the chemical process.

**Table 1:** Pseudo-first-order and pseudo-second-order model parameters for GOMO.

Kinetic model	*T* (°C)	*c*_0_ (mg/l)	*Q*_e_ (mg/g)	*k*_1_ (g/(mg min)	*k*_2_ (g/(mg min)	*R* ^2^
Pseudo-first-order	25	10	0.35	0.0198	—	0.9443
Pseudo-second-order	25	10	19.92	—	0.2520	1.0000

#### Influence of initial U(VI) concentration on sorption capacity

**Figure 8:**
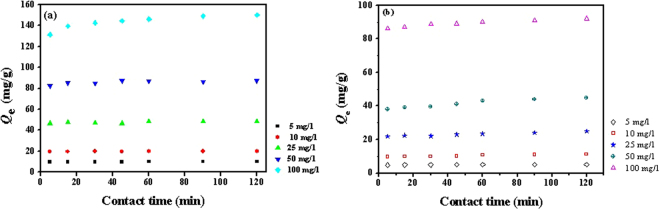
Influence of initial uranium concentration on sorption capacity of GOMO (**a**) and GO (**b**). pH = 4.0, adsorbent dosage = 0.5 g/l, T = 298 K.

The influence of different U(VI) concentrations on the sorption capacity of GOMO is shown in Fig. 8. GOMO had a low sorption capacity at low initial concentrations of <10 mg/l, which was consistent with the findings of Wang *et al*.^22^. However, the sorption capacity of GOMO was significantly elevated and rapidly reached adsorption equilibrium when initial U(VI) concentration increased. The sorption capacity reached above 150 mg/g when the concentration was 100 mg/l. Therefore, it is seen from Fig. 8 that the initial concentration played a significant role in driving U(VI) to adsorb onto the surface of GOMO. Meanwhile, the adsorption capacity presented a very steady trend due to the active sites of GOMO being very rapidly occupied by U(VI) ions and reaching sorption saturation.

#### Influence of ionic strength on sorption efficiency

**Figure 9:**
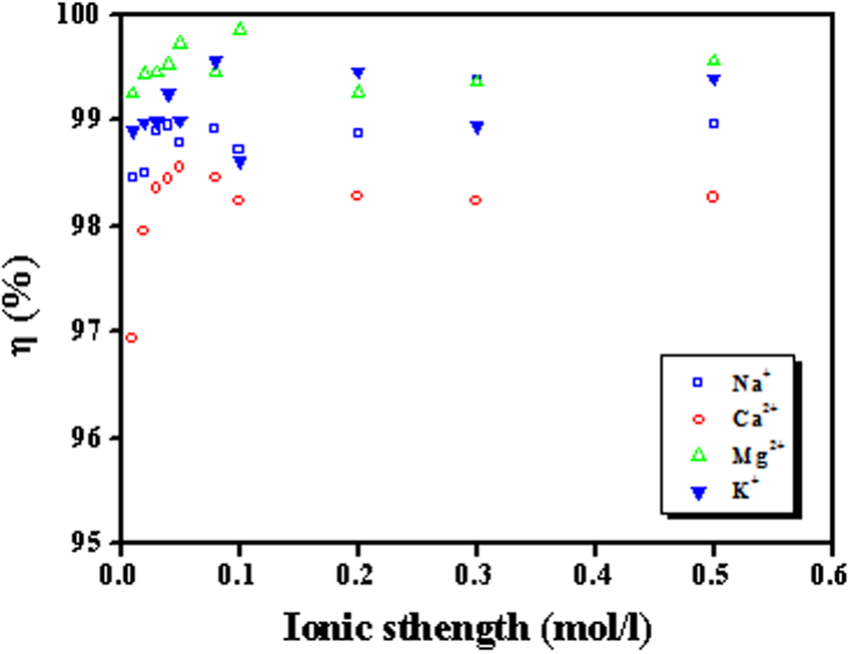
Influence of ionic strength on sorption efficiency of GOMO. pH = 4.0, *C*_0_(U) = 10 mg/l, adsorbent dosage = 0.5 g/l, T = 298 K, t = 30 min.

Currently, some important cations (e.g., Na^+^, K^+^, Ca^2+^ and Mg^2+^) are present universally with relatively high concentrations (>0.2 M) in nuclear waste liquid. However, the ionic-exchange technique depends on high ionic strength and is only suitable for the treatment of uranium-bearing wastewater with the concentration below 0.01 M^23^. To present the applicability of GOMO in the solutions containing varying ionic concentrations (0.01~0.5 M), the effect of ionic strength (Na^+^, K^+^, Ca^2+^ and Mg^2+^) on the removal efficiency of uranium was tested. The results are presented in Fig. 9. The results showed that ionic strength did not significantly influence the U(VI) adsorption. Therefore GOMO proved to be a promising adsorbent for nuclear waste liquid even in the presence of very high salinity. The adsorption phenomena generally include inner sphere complexation, outer sphere complexation and ion exchange^24^. Inner sphere complexation is evidently influenced by pH whereas ionic strength will affect outer sphere complexation and ion exchange^25^. Consequently, the uranium adsorption by GOMO was considered to be inner sphere complexation, which was similar with the ref.^26,27^.

### Adsorption isotherm

**Figure 10:**
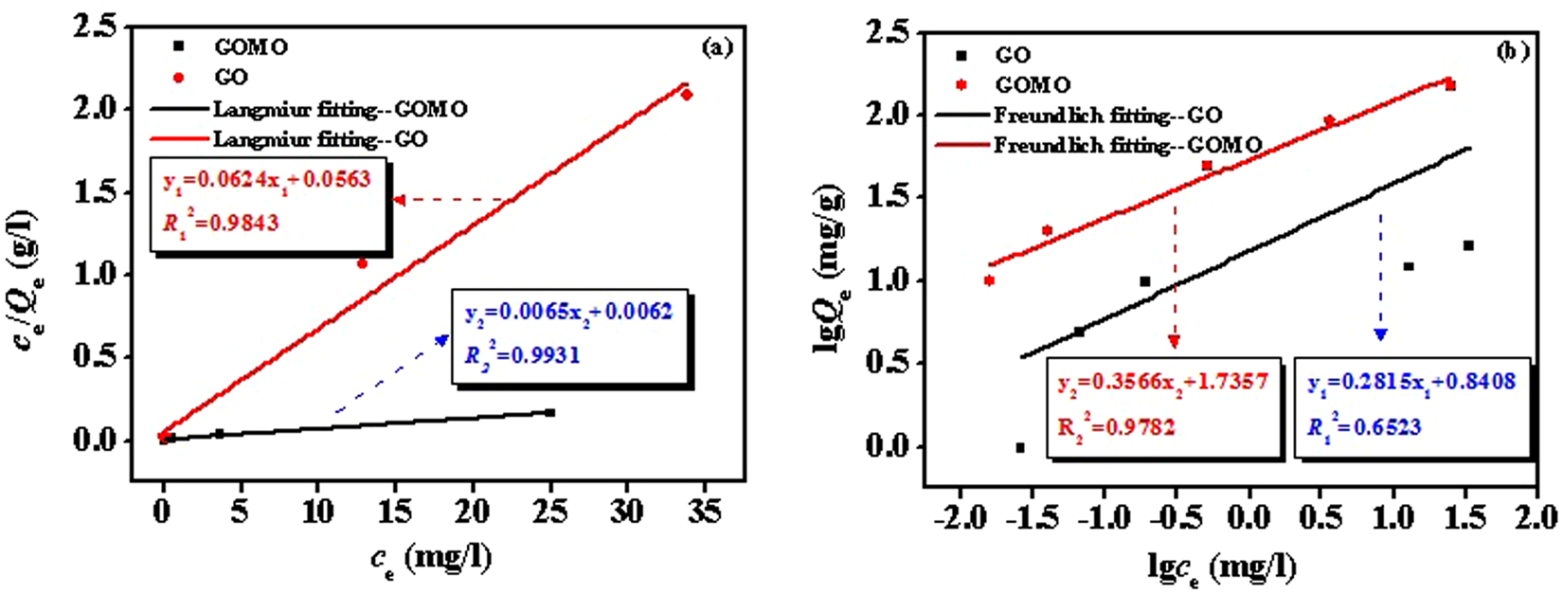
Fitting lines of Langmuir (**a**) and Freundlich (**b**) isotherm models of GO and GOMO.

Figure 10 shows that linear fit of Langmuir and Freundlich isotherm models of U(VI) adsorbed by GO and GOMO. The Langmuir and Freundlich isotherm parameters are given in Table 2. The adsorption of GOMO and GO fitted Langmuir isotherm model well which indicated that the adsorption process was a monolayer uptake of U(VI) on GOMO and GO. Comparison with GO (*Q*_m_ = 16.03 mg/g) shows that the maximum adsorption capacity of GOMO (*Q*_m_ = 153.85 mg/g) was improved significantly. Meanwhile, l/*n* was 0.2815 (0.1 < 1/*n* < 0.5), suggesting that the U(VI) adsorption on the GOMO was favourable.

**Table 2:** Sorption parameters for Langmuir and Freundlich isotherm models.

Adsorbent	*Q*_m_ (mg/g)	Langmuir	*R* ^2^	*n*	Freundlich	*R* ^2^
		*k*_L_ (l/mg)			*k*_F_ (mg^1−n^ l^n^/g)	
GO	16.03	1.11	0.9843	3.55	6.93	0.6523
GOMO	153.85	1.05	0.9931	2.80	54.41	0.9782

A comparison of *Q*_m_ of GO and GOMO in this study and other adsorbents at 298 K is presented in Table 3. It is clear that compared to other listed absorbent, *Q*_m_ of GOMO was excellent, suggesting that GOMO shows promising potential for the uranium-bearing wastewater treatment.

**Table 3:** Comparison of the maximum adsorption capacity of various sorbents for uranium at 298 K.

Sorbents (ref.)	*Q*_m_ (mg/g)	Sorbents (ref.)	*Q*_m_ (mg/g)
AMGO^34^	123.40	Al_2_O_3_ nanofibres^35^	204.10
Fe-SC^36^	148.99	Fe_3_O_4_@TiO_2_^37^	118.80
Fe_3_O_4_/GO^38^	69.49	Layered double oxides/carbon^39^	354.2
Magnetic cucurbit[6]uril/GO^40^	122.50	g-C_3_N_4_@Ni-Mg-Al-LDH^41^	99.7
Oxime-grafted CMK-5^42^	62.00	*l*-C_3_N_4_/PDA/PEI_3_^43^	60.51
Polyacrylamide–hydroxyapatite^44^	0.95	TiO_2-x_^45^	65
Polypyrrole^46^	87.72	CaTiO_x_; CaAlO_x_^47^	241.7; 258.29
Amidoxime modified Fe_3_O_4_@SiO_2_^48^	105.50	Titanate^49^	358
Modified silica gel^50^	90.30	CNFs^51^	125
Birnessite-modified pine biochar^52^	47.05	p-AO/ CNFs; c-AO/CNFs^53^	588.24; 263.18
Talc^54^	41.60	RUB-15^55^	152
MnO_2_–Fe_3_O_4_–RGO^56^	108.70	GO-CS-P^57^	779.44
SA/CMC-Ca-Al^58^	101.76	Ca/Al-LDH@CNTs^59^	382.9
Phosphate-modified pine wood sawdust^60^	74.10	GO (Present study)	16.03
GOMO (Present study)	153.85		

### Thermodynamic studies

The thermodynamic parameters Δ*G*°, Δ*H*° and Δ*S*° were studied from 298 K to 333 K (sorbent dose = 0.5 g/l, *C*_0_(U) = 10 mg/l, *V* *=* 20 ml, *t* *=* 30 min, pH = 4.0) and were calculated using Eqs (1–3)^28^:

1 $${K}_{d}=\frac{{c}_{ad}}{{c}_{e}}$$

2 $${\rm{In}}\,{{K}}_{d}=-\,\frac{{\rm{\Delta }}{{H}}^{0}}{RT}+\frac{{\rm{\Delta }}{{S}}^{0}}{R}$$

3 $${\rm{\Delta }}{G}^{0}={\rm{\Delta }}{H}^{0}-T{\rm{\Delta }}{S}^{0}$$

**Figure 11:**
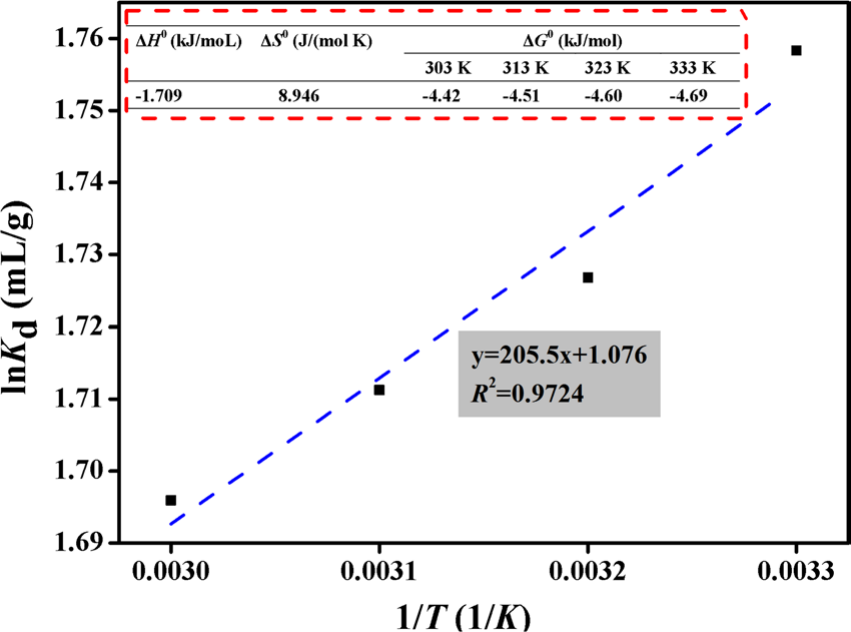
Plot of ln*K*_d_ versus 1/*T* for U(VI) adsorption onto GOMO. pH = 4.0, *C*_0_(U) = 10 mg/l, adsorbent dosage = 0.5 g/l, T = 303 K, 313 K, 323 K and 333 K, t = 30 min. Inset showFigus the thermodynamic parameters of U(VI) adsorption on GOMO.

Where *R* (8.314 J/(mol·K)) is the universal gas constant, *K*_d_ is the sorption equilibrium constant and *T* (K) is the absolute temperature. The plot of ln*K*_d_ versus 1/*T* for U(VI) adsorption onto GOMO is presented in Fig. 11, and the insert shows the thermodynamic parameters. The negative value of Δ*H*° (−1.709 kJ/mol) reflected the fact that adsorption is an exothermic reaction. The positive Δ*S*^0^ and negative Δ*G*^0^ indicated that GOMO was a spontaneous adsorption process. Furthermore, the low absolute values of Δ*G*^0^ and Δ*H*^0^ indicated that the sorption process was physisorption^29^.

### Regeneration and reuse

**Figure 12:**
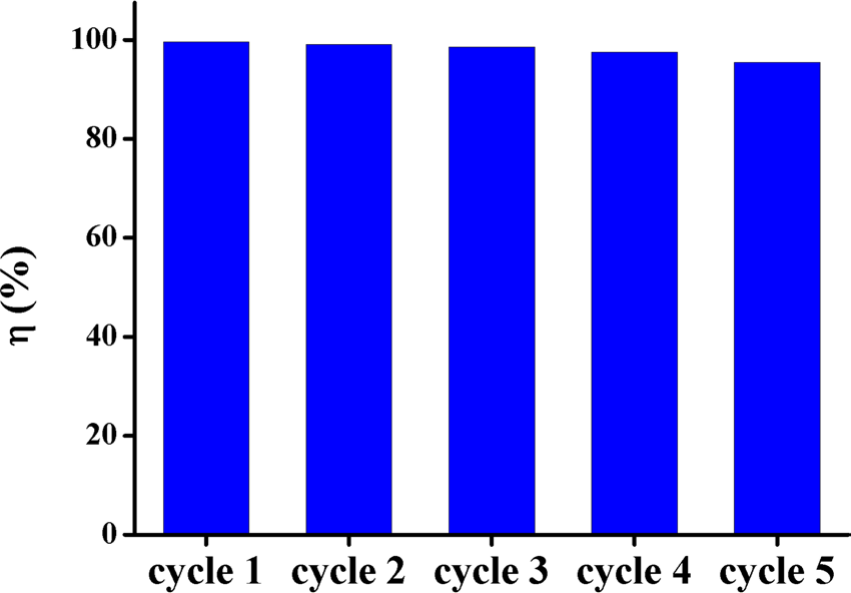
Reusability results for GOMO over five cycles using 3 M HNO_3_ as regenerant. pH = 4, *C*_0_(U) = 10 mg/l, *T* = 298 K, adsorbent dosage = 0.5 g/l.

Regeneration and cost saving have been very important for the wastewater treatment process. The reuse of GOMO was examined when nitric acid (3 M) was used as the regenerant. The results are presented in Fig. 12. The removal rate of U(VI) could reach 95.45% after five cycles. The results proved that GOMO could be used repeatedly for U(VI) adsorption, and the removal rate of uranium decreased only slightly through five cycles.

## Materials and Methods

### Materials

Natural graphite was purchased from the Aladdin Chemistry Co. (Shanghai, China). All other chemicals were analytical grade and used without any purification.

### Preparation of GOMO

GO were prepared from natural graphite by the modified Hummers method^14^. GOMO was prepared using GO and KMnO_4_ in the acidic conditions under ultrasonic irradiation. Briefly, GO (0.6 g) in the 40 ml deionized water was sonicated in an ultrasonic bath (Branson 2510, USA) for 2 h in a 250-ml beaker. Then, KMnO_4_ (0.9 g) and concentrated HCl (2 ml) were added to the suspension of GO. The resulting mixture was sonicated in an ultrasonic bath for 30 min at 60 °C. The precipitates were centrifuged and washed with deionized water and alcohol. Finally, the products were collected and dried at 50 °C under vacuum.

### Characterizations

The structures of GO and GOMO were characterized using FTIR spectroscopy (Bruker VERTEX 70, Germany), raman shift spectroscopy (Bruker VERTEX 70, Germany) and X-ray diffraction patterns (2700 model, China). Scanning electron microscopy (SEM) images of GO and GOMO were obtained using an electron microscope (Helios 600i, Japan).

### Adsorption experiments

Standard solutions of uranium (100 μg/ml) were purchased from Chemical Engineering and Metallurgy Research Institute (Beijing, China). The pH of the uranium solutions (20 ml) was adjusted with HCl and NaOH by a pH meter (pHS-25 model, China). The adsorbent was then added to the uranium solution, which was shaken on a shaker (Kangshi, China). After filtration, the residual uranium concentrations were measured by a micro-quantity uranium analyser (MUA model, China). The removal rate η (%) and adsorption capacity at equilibrium *Q*_e_ (mg/g) of uranium were calculated using Eqs (4) and (5), respectively:

4 $$\eta ( \% )=\frac{{c}_{0}-{c}_{t}}{{c}_{0}}\times 100$$

5 $${Q}_{e}(mg/g)=\frac{({c}_{0}-{c}_{e})V}{W}$$

where *c*_0_ and *c*_*t*_ (mg/l) are initial concentration and concentration at time *t* of U(VI), respectively, *c*_e_ (mg/l) is equilibrium concentration of U(VI), *W* (g) is the adsorbent mass, and *V* (l) is the solution volume.

### Adsorption isotherm

The Langmuir and Freundlich sorption isotherms of GO and GOMO were analysed with different uranium concentrations (5–100 mg/l, *T* = 298 K, sorbent dosage = 0.5 g/l, t = 30 min). The Langmuir isotherm equation^30^ assuming the monolayer adsorption process is expressed as follows:

6 $$\frac{{c}_{e}}{{Q}_{e}}=\frac{1}{{Q}_{m}{K}_{L}}+\frac{{c}_{e}}{{Q}_{m}}$$

7 $${R}_{L}=\frac{1}{1+{K}_{L}{c}_{0}}$$

The Freundlich isotherm^31^ is expressed using Eq. (8).

8 $${\rm{lg}}\,{Q}_{e}=\,{\rm{lg}}\,{K}_{F}+\frac{1}{n}\,{\rm{lg}}\,{{c}}_{e}$$

where *c*_e_ (mg/l) is the concentration at equilibrium, *Q*_e_ (mg/g) is the adsorption capacity at equilibrium, *Q*_m_ (mg/g) is the maximum adsorption capacity, *K*_L_ (l/mg) and *K*_F_ (mg^1−n^ l^n^/g) are Langmuir and Freundlich constant, respectively, and *n* is the Freundlich exponent.

### Adsorption kinetics

The pseudo-first-order^32^ and pseudo-second-order rate equation^33^ are commonly applied to describe the sorption rate and kinetic mechanism, and are expressed using Eqs (9) and (10), respectively:

9 $${\rm{lg}}({Q}_{e}-{Q}_{t})=\,\mathrm{lg}\,{Q}_{e}-\frac{{k}_{1}}{2.303}t$$

10 $$\frac{t}{{Q}_{t}}=\frac{1}{{k}_{2}{Q}_{e}^{2}}+\frac{t}{{Q}_{e}}$$

Where *k*_1_ (g/(mg·min)) is Lagergren rate constant, *Q*_e_ (mg/g) is the adsorption capacity at equilibrium, *Q*_t_ (mg/g) is the adsorption capacity at time *t*, and *k*_2_ (g/(mg·min)) is the pseudo-second-order rate constant.

### Regeneration and reuse of GOMO

After adsorption experiments, the obtained U-loaded GOMO was rinsed and washed with 3 M HNO_3_ solution and deionized water until no U(VI) was detected in the solution. Then, the regenerated and dried GOMO was reused for further adsorption experiments.

## Conclusions

A composite adsorbent GOMO was successfully synthesized by a facile ultrasonic radiation method. GOMO showed high adsorption efficiency for uranium from aqueous solutions at pH = 4.0–6.0. pH significantly influenced the sorption of U(VI) onto GOMO. For the uranium solution of 10 mg/l, the removal of U(VI) reached near completion within 20 min under the sorbent dosage of 0.5 g/l. Compared to GO (*Q*_m_ = 16.03 mg/g), *Q*_m_ of GOMO was improved significantly and reached 153.85 mg/g. GOMO proved to be a promising sorbent for uranium-bearing nuclear wastewater with a high salinity. The sorption data fitted the Langmuir isotherm model and pseudo-second order model well. The adsorption of GOMO for U(VI) proved to be the chemical sorption process. Thermodynamic investigation revealed that U(VI) adsorption onto GOMO was spontaneous and exothermic. The reuse experiments were carried out using 3 M HNO_3_, and the sorption efficiency of the regenerated GOMO had only a little decrease after five cycles.
